# Numerical Simulation and Experimental Validation of Hybrid Injection Molded Short and Continuous Fiber-Reinforced Thermoplastic Composites

**DOI:** 10.3390/polym13213846

**Published:** 2021-11-07

**Authors:** Patrick Hirsch, Marianne John, Daniel Leipold, André Henkel, Sylvia Gipser, Ralf Schlimper, Matthias Zscheyge

**Affiliations:** Fraunhofer Institute for Microstructure of Materials and Systems IMWS, 06120 Halle (Saale), Germany; marianne.john@imws.fraunhofer.de (M.J.); daniel.leipold@imws.fraunhofer.de (D.L.); andre.henkel@imws.fraunhofer.de (A.H.); sylvia.gipser@imws.fraunhofer.de (S.G.); ralf.schlimper@imws.fraunhofer.de (R.S.); matthias.zscheyge@imws.fraunhofer.de (M.Z.)

**Keywords:** numerical simulation, hybrid injection molding, continuous fiber-reinforced thermoplastics

## Abstract

In-situ thermoforming and overmolding of continuous fiber-reinforced thermoplastic composites by hybrid injection molding enables the mass production of thermoplastic lightweight structures with a complex geometry. In this study, the anisotropic mechanical behavior of such hybrid injection molded short and continuous fiber-reinforced thermoplastics and the numerical simulation of the resulting mechanical properties under flexural loading were investigated. For this, the influence of the volume flow rate between 25 and 100 cm^3^/s during injection molding of a PP/GF30 short fiber-reinforced overmolding material was studied and showed a strong effect on the fiber orientation but not on the fiber length, as investigated by computer tomography and fiber length analysis. Thus, the resulting anisotropies of the stiffness and strength as well as the strain hardening investigated by tensile testing were considered when the mechanical behavior of a hybrid test structure of short and continuous fiber-reinforced thermoplastic composites was predicted by numerical simulations. For this, a PP/GF60 and PP/GF30 hybrid injection molded test structure was investigated by a numerical workflow with implemented injection molding simulation data. In result, the prediction of the mechanical behavior of the hybrid test structure under flexural loading by numerical simulation was significantly improved, leading to a reduction of the deviation of the numerically predicted and experimentally measured flexural strength from 21% to 9% in comparison to the isotropic material model without the implementation of the injection molding data.

## 1. Introduction

The use of thermoplastic fiber-reinforced composites as high-performance materials for lightweight structures has been going on for several decades. Continuous fiber-reinforced thermoplastics (TPC) processed by pultrusion unidirectional fiber-reinforced tapes are regarded as the material class with the highest lightweight potential in many applications combining high mechanical properties and economical manufacturing processes [1]. Furthermore, TPCs enable a load adapted structural design by orientation of the fiber direction to the load path of the part application [2]. The specific design of such TPCs is based on the constitutive equation theory of laminates [3,4,5]. In doing so, numerical simulations with adjusted material models do allow the prediction of the rate dependent non-linear mechanical behavior of complex shaped parts made out of TPCs [6]. Recent technology developments made in-situ thermoformed and overmolded laminate structures of TPCs available by hybrid injection molding, which enables the mass production of thermoplastic lightweight structures with a complex geometry and a high degree of functional integration [7]. The process flow of this technology is comparable to the already implemented process flow of continuous fiber-reinforced thermoplastics consisting of textile fiber components and consist of the heating, hot handling and thermoforming of the TPC with subsequent overmolding in the same mold [8,9]. Typical applications of such thermoplastic lightweight structures are automotive parts with large-scale production [10]. However, the implementation of this approach for reliable lightweight structures is strongly dependent on the ability to precisely predict the mechanical behavior of the processed parts in the application, which is usually carried out by reverse engineering and numerical simulation techniques [11,12]. In doing so, structural analysis of hybrid injection molded parts must include the anisotropic mechanical behavior of the TPC as well as of the long or short fiber-reinforced overmolding material. Since the local fiber orientation of the TPC laminate is adjusted and therefore known, the major focus to improve the prediction accuracy of the structural properties has to be on the correct consideration of the microstructure of the overmolding material and its anisotropic behavior. In here, the local fiber orientation is strongly dependent on the part geometry, raw material constitutions and processing conditions but can be numerically simulated and implemented in the structural analysis by a multi-scale methodology [13,14]. However, deviations between the predicted mechanical behavior by the numerical simulations and the experimental results, especially for complex part geometries, are still a problem and can be attributed to imprecise mapping of the local fiber orientation of the overmolding material from the injection molding simulation to the structural analysis [14]. Additionally, the non-isothermal formation of the boundary between the TPC and the overmolding material, e.g., by simulation of the molecular dynamics at the interface, has to be considered in hybrid processes to reduce the error between the simulation and the real structural behavior [15,16].

Thus, a more advanced workflow by connection of the injection molding simulation, the material modelling and the structural analysis of the processed part has to be implemented for precise prediction of the mechanical behavior of hybrid injection molded short and continuous fiber-reinforced thermoplastic composite parts. However, the implementation of the local fiber orientations of the overmolding material from the injection molding simulation in the structural analysis is actually not a fully automated process, e.g., impaired by different mesh types. In result, underprediction or overprediction of the local stiffness and strength of the overmolding material depending upon the fiber orientation and direction of loading does occur. In this study, the numerical simulation of a hybrid injection molded continuous fiber-reinforced thermoplastic composite test structure was carried out by a new numerical workflow. This new workflow allows the automated implementation of the local fiber orientation from injection molding simulations to structural analysis by finite element modelling and therefore a more precise consideration of the local anisotropic mechanical behavior of the overmolding material including its hardening behavior. Validation of the predicted mechanical behavior of processed hybrid test structures under flexural loading was carried out by experimental analysis. Additionally, comparison of isotropic and anisotropic structural simulations was carried out to investigate prediction accuracy of the new workflow especially at higher deformations in the flexural tests.

## 2. Materials and Methods

### 2.1. Materials

#### 2.1.1. Continuous Fiber-Reinforced Thermoplastic Composites

TPC laminates made of unidirectional glass fiber-reinforced polypropylene tapes (PP/GF60-UD, Plytron GN 638T, Elekon, Luzern, Switzerland) were used for the experimental investigations. Important properties of the used tapes are shown in Table 1. The manufactured laminates consisted of 8 layers with a symmetrical and balanced 0°/90°-lay-up (0°, 90°, 90°, 0°, 0°, 90°, 90°, 0°). In result, TPC laminates with total dimensions of 396 mm × 120 mm × 2 mm were used as laminate inserts for the hybrid injection molding process.

#### 2.1.2. Short Fiber-Reinforced Thermoplastic Composites

Overmolding of the TPC laminates was carried out with a short glass fiber-reinforced polypropylene compound (PP/GF30, Hostacom G3 N01 L, Lyondell Basell, Rotterdam, Netherlands) to investigate the resulting mechanical properties of the hybrid injection molded test structures. The compound was used in pellet form with a fiber mass content of 30 wt% and was dried before injection molding at 80 °C for 4 h. Important properties of the overmolding material are shown in Table 2.

### 2.2. Experimental Work

#### 2.2.1. Production of Test Structures

##### Injection Molding of Short Fiber-Reinforced Thermoplastic Composites

**Figure 1:**
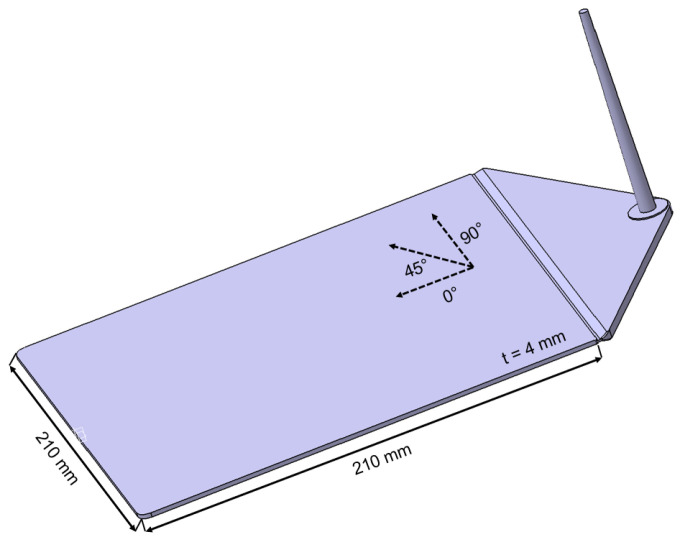
Geometry of the injection molded test plates made of PP/GF30 short fiber-reinforced thermoplastic composite.

**Figure 2:**
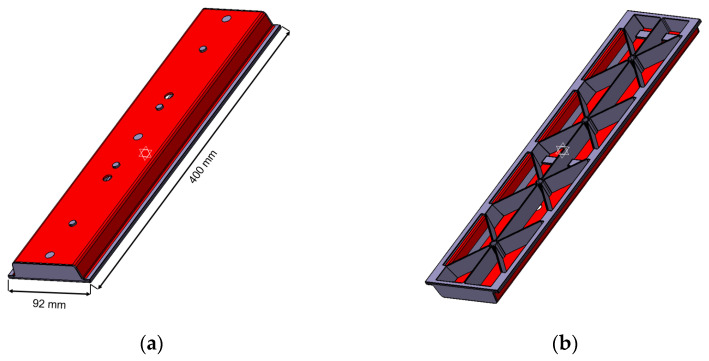
Geometry of the hybrid injection molded thermoplastic composite bending test structure with the PP/GF60 continuous fiber-reinforced thermoplastic composite in red and the PP/GF30 short fiber-reinforced thermoplastic composite in grey: (**a**) top and (**b**) bottom.

An industrial injection molding machine with a maximum clamping force of 200 t (KM200-1000C2, KraussMaffei, Munich, Germany) was used for the experimental processing tests and its influence to the anisotropic mechanical properties of the short glass fiber-reinforced thermoplastic (see Table 3). Thus, test plates with the dimensions of 210 mm × 210 mm × 4 mm were produced from the PP/GF30 compound with a melt temperature of 230 °C and a mold temperature of 40 °C. To investigate the influence of the flow profiles and the corresponding shear stresses on the resulting mechanical properties, the experiments were carried out by varying the volume flow rate between 25, 50 and 100 cm^3^/s. The subsequent analysis of the samples was carried out by means of fiber length and fiber orientation analysis as well as tensile testing of standard test specimens machined out in 0°, 45° and 90° related to the flow direction, as shown in Figure 1. In result, nine different sets of test specimens were analyzed regarding their mechanical properties. Labeling of those sets can be found in Table 3. Additionally, test structures with the geometry shown in Figure 2 were made of the hybrid injection molded thermoplastic composites for comparative experimental and numerical investigations.

##### Hybrid Injection Molding of Short and Continuous Fiber-Reinforced Thermoplastic Composites

**Figure 3:**
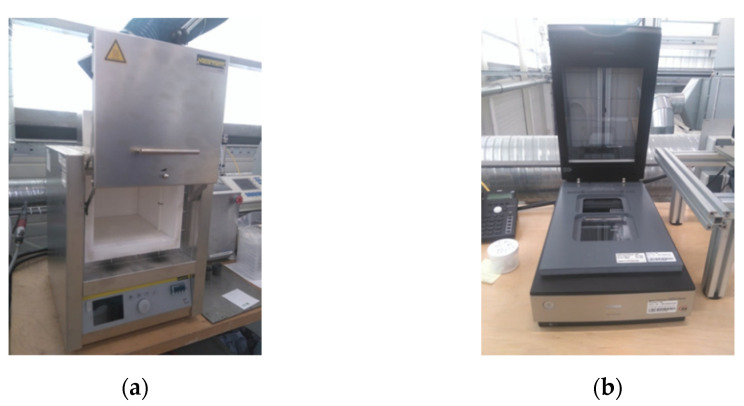
Experimental equipment used for fiber length analysis of the PP/GF30 short fiber-reinforced thermoplastic composite: (**a**) electric furnace and (**b**) photo scanner.

For the production of the hybrid injection molded short and continuous fiber-reinforced thermoplastic structures an industrial injection molding machine with a maximum clamping force of 200 t (KM200-1000C2, KraussMaffei, Munich, Germany) was used, additionally equipped with an infrared heating station and automation robot-gripper system. The whole process had a cycle time of 120 s and consisted of the infrared heating of the TPC laminates to a temperature of 215 °C, the hot handling of the heated laminates and fixation in the mold by the robot and the subsequent thermoforming as well as overmolding with the short fiber-reinforced thermoplastic composite material (using melt temperature of 210 °C and a mold temperature of 45 °C). The volume flow rate during the injection step was set to 50 cm^3^/s and the central injection point of the complex hybrid structure was used to avoid any weld lines in the overmolded short fiber-reinforced composite material. A schematic depiction of the geometry of the produced hybrid structures is shown in Figure 3.

#### 2.2.2. Morphological Analysis

##### Fiber Length Analysis of Short Fiber-Reinforced Thermoplastic Composites

The fiber length analysis of the test specimens was performed in two steps. First, the test specimens were pyrolyzed at 600 °C for 3 h in an electric furnace (LT 5/12, Nabatherm, Lilienthal, Germany) to separate the fibers from the matrix. Subsequently, the fibers were suspended in a water solution and then scanned with a photo scanner (Perfection V800 Dual Lens, Epson, Suwa, Nagano, Japan) to determine their fiber length distribution. The scanned images were analyzed using FIVER software to acquire the number of fibers and the length distribution for each sample.

##### Fiber Orientation Analysis of Short Fiber-Reinforced Thermoplastic Composites

**Figure 4:**
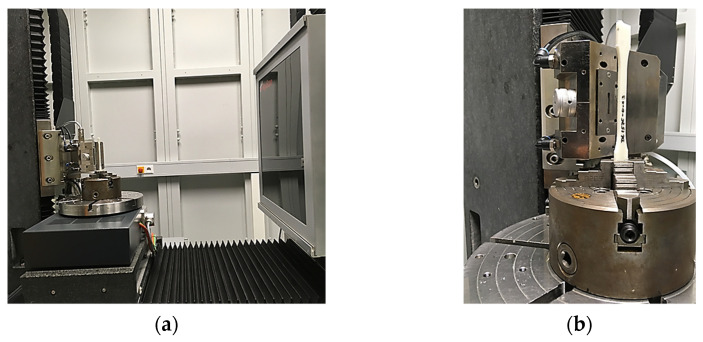
Experimental set up used for fiber orientation analysis of the PP/GF30 short fiber-reinforced thermoplastic composite: (**a**) computed tomography system and (**b**) test specimen clamped on turntable.

The fiber orientation analysis of the test specimens was carried out by computed tomography (CT). For this, each test specimen was clamped on the turntable of the CT system (RayScan 200E, RayScan Technologies GmbH, Meersburg, Germany) and X-ray projections were taken during a full circulation of 360°, using 600 projections per second with a voltage of 70 kV and a current of 130 µA (see Figure 4). The resulting 3D image data set was analyzed using VGStudio analysis software (VGStudio MAX 2.2, Volume Graphics GmbH, Heidelberg, Germany).

#### 2.2.3. Mechanical Testing

##### Tensile Test of Short Fiber-Reinforced Thermoplastic Composites

**Figure 5:**
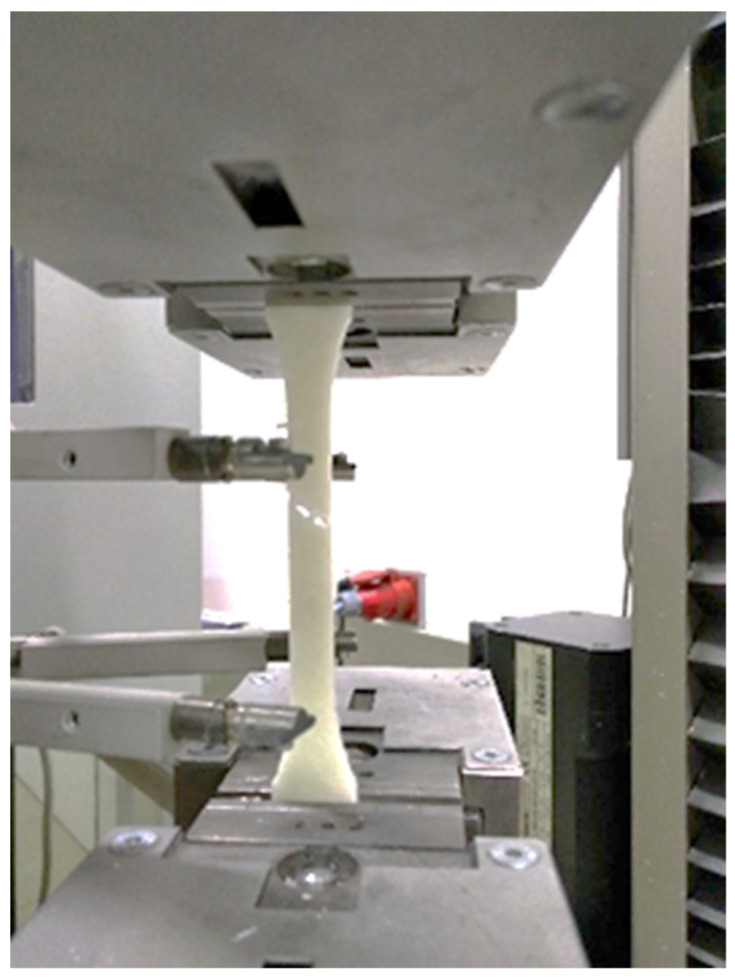
Tensile test according to ISO 527 on machined out standard test specimens of the PP/GF30 injection molded short fiber-reinforced thermoplastic composite.

Investigation of the anisotropic mechanical properties of the injection molded test plates of the PP/GF30 compound was carried out by tensile tests on machined out 1A test specimens according to ISO 527 at 24.5 °C with a universal test machine (Z050, Zwick Roell, Ulm, Germany) as shown in Figure 5. The measurement of the tensile modulus was carried out at 1 mm/min test speed, while the tensile strength was tested at 10 mm/min. The test specimens were machined out from the injection molded test plates in 0°, 45° and 90° related to the flow direction.

##### Flexural Test of Hybrid Injection Molded Thermoplastic Composites

**Figure 6:**
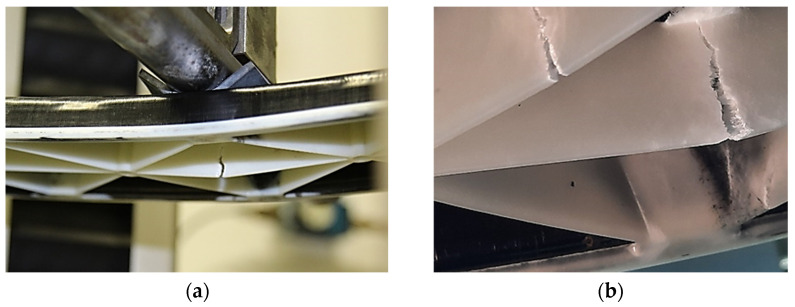
Three-point flexural test of the PP/GF60 and PP/GF30 hybrid injection molded thermoplastic composite test structure: (**a**) test set-up and (**b**) failure of ribs as a result of the bending load.

Three-point flexural tests of the hybrid injection molded thermoplastic composites were performed according to DIN EN ISO 14125, recommended for bending of continuous fiber-reinforced plastics, at 22.8 °C (room temperature, RT) with a universal testing machine (Z050, Zwick Roell, Ulm, Germany). A support spacing of 360 mm was used for the tests, while the supports had a diameter of 30 mm. The continuous deformation speed of the tests was set to 2 mm/min and the test was carried out until failure of the structure occurred. As can be seen in Figure 6, the failure was located in the rip area consisting of short fiber-reinforced thermoplastic composite overmolding material. The force of the machine was recorded as well as the displacement by extensiometer under the middle of the specimen (center deflection).

### 2.3. Numerical Simulations

#### 2.3.1. Theoretical Background

Describing the correct mechanical behavior of hybrid thermoplastic composites through finite element modeling simulations has been traditionally a challenge because of the complex anisotropic, inhomogeneous and multiscale nature of these materials. Hybrid TPCs combine unidirectional or woven fabrics made of continuous fibers and thermoplastic matrix (stacked, heated and compressed before to laminates) with a short fiber reinforced thermoplastic material via injection molding (overmolding). The resulting TPC hybrid component is a continuous structure that has the complex and three-dimensional shape of an injection molded part and the structural backbone of a continuous composite laminate.

The most common approach in finite element simulations of composite materials is based on macroscale phenomenological modelling, where layered shells, layered-solids, stacked solid elements and stacked or layered continuum shells are used to represent the laminate. The specific material properties of each laminate typically are obtained through experimental investigations and the application of the classical laminate theory [17]. However, such a phenomenological modelling technique has a limited applicability only to continuous fiber-reinforced composites with anisotropic but local nearly homogenous properties. Thus, multiscale techniques are required to capture the complex microstructures in hybrid injection molded short and continuous fiber-reinforced thermoplastic composites [18,19]. Nevertheless, multiscale techniques have traditionally encountered a limited adoption in modeling of the mechanical behavior of composites because of the large requirements in terms of computing capacity for such simulations. Thus, a variety of acceleration techniques have been developed over the years to solve the computing capacity issues related to the solution of finite element static problems involving two-scale homogenization approaches (FE2 analyses) [20], among them reduced order models [21,22], the (non-uniform) transformation field analysis [23], response surface models [24,25] and machine learning approaches such as neural networks [26,27]. However, while these homogenization techniques can be used to describe the material behavior dependent on the specific local microstructure, further modeling and numerical challenges arise after the onset of failure (e.g., cracking, fiber bridging, and delamination mechanisms) occurring in the composite component at higher deformations [28,29,30]. Capturing the details of such nonlinear post failure behavior is still the subject of different research investigations and is neglected in this work.

#### 2.3.2. Overview of the Workflow

**Figure 7:**
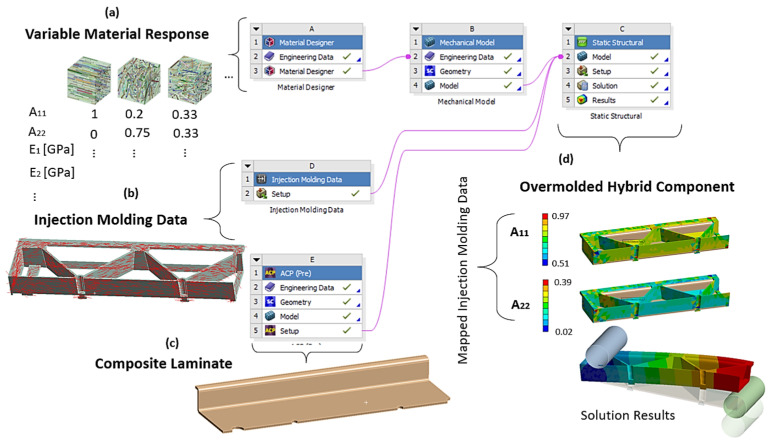
Workflow of the multiscale model in ANSYS Mechanical using the Material Designer (MD): (**a**) creation of the material response as a function of the microstructure by an interpolation grid with attached elastic and plastic parameters, (**b**) import of injection molding data, i.e., the second order orientations tensors from the injection molding software, (**c**) modeling of the ply-based continuous fiber-reinforced TPC laminate and (**d**) mapping of the injection molding data to the short fiber-reinforced parts and bonding to the continuous fiber-reinforced laminate.

**Figure 8:**
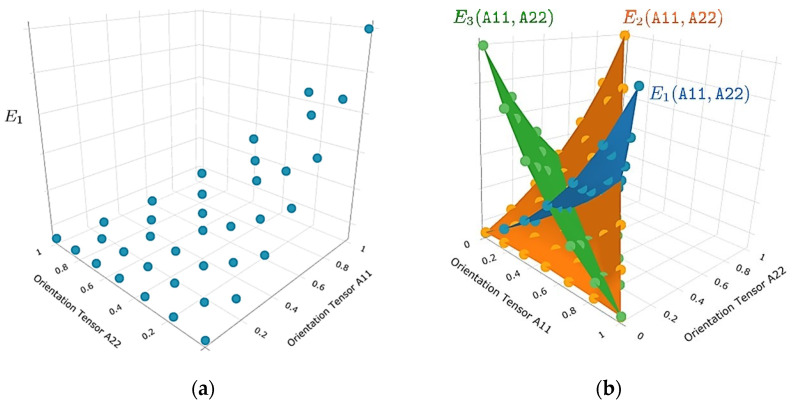
Visualization of the properties of generated variable material response with respect to the axes of principal orientations: (**a**) interpolation grid for E1 and (**b**) interpolation grids and interpolation surfaces for E1, E2 and E3 as functions of principal orientations A11 and A22. The user is given the possibility to generate analogous plots for the other 6 homogenized elastic parameters of the orthotropic behavior in the coordinate system defined by the principal axes of 2nd order orientation tensors.

The numerical workflow of the used multiscale approach for hybrid composites is shown in Figure 7. The relevant elastic properties and the mechanical behavior of continuous fiber-reinforced laminates were determined experimentally. The tool Material Designer (MD) in ANSYS Workbench (ANSYS 2021R1, Ansys Inc., Canonsburg, PA, USA) was used uniquely for capturing the material response of the short fiber-reinforced overmolding material of the hybrid composite component. Within MD, for a number of sample orientations, an effective elastic-plastic model was derived by a mean-field homogenization method based on the Mori-Tanaka formulation [31]. Considering symmetry arguments, the fiber orientation tensor was reduced to a triangle in the 2-dimensional space given by the eigenvalues of the orientation tensor, as shown in Figure 8. Then, linear interpolation on the fiber orientation triangle was used to compute the elastic-plastic properties for each imported fiber orientation state (elastic moduli and Hill’s yield strengths of the plastic response for the material directions in tensorial listing). In the analysis carried on for this work, the combination of the Mori-Tanaka method with orientations averaging based on the closure approximation by Cintra and Tucker [32] were used for the derivation of elastic properties. For calculation of the plastic response, an orientation-dependent anisotropic Hill yield criterion was combined with an isotropic hardening model following the empirical exponential model suggested by Voce and anisotropic yield surface [33,34]. The needed stress-strain values were derived from uniaxial tension experiments with two types of test specimens: in flow direction and also perpendicular to the flow direction from the injection molded plates of the short fiber-reinforced composite material used for overmolding. The fiber orientation field of these test specimens were obtained numerically by injection molding simulations. In result, two test curves consisting of the uniaxial true stress versus true plastic strain, from tensile specimens cut out flow direction (0°) and perpendicular to it (90°), were obtained and subsequently fitted to determine the non-linear isotropic hardening model parameters of the short fiber-reinforced composite material. This variable material response as a function of the fiber orientations was then combined with the imported local fiber orientation in each element from the injection molding simulation and transferred to the mechanical solver.

#### 2.3.3. Numerical Simulation of Injection Molding

The numerical simulations for the injection molding of the test plates with the PP/GF30 compound as well as the hybrid injection molding parts in combination with TPC laminate inserts were carried out using Moldex3D software (Moldex3D 2021R1OR, CoreTech System Co., Ltd., Zhubei, Taiwan). Here, based on the gate and cooling system as well as the mold cavity geometry, a model with a volume mesh was built up. The simulation parameters were derived from the processing parameters of the real injection molding processes. The thermodynamic and rheological material parameters of the examined PP/GF30 short fiber-reinforced thermoplastic composite were taken from the Moldex3D database.

**Figure 9:**
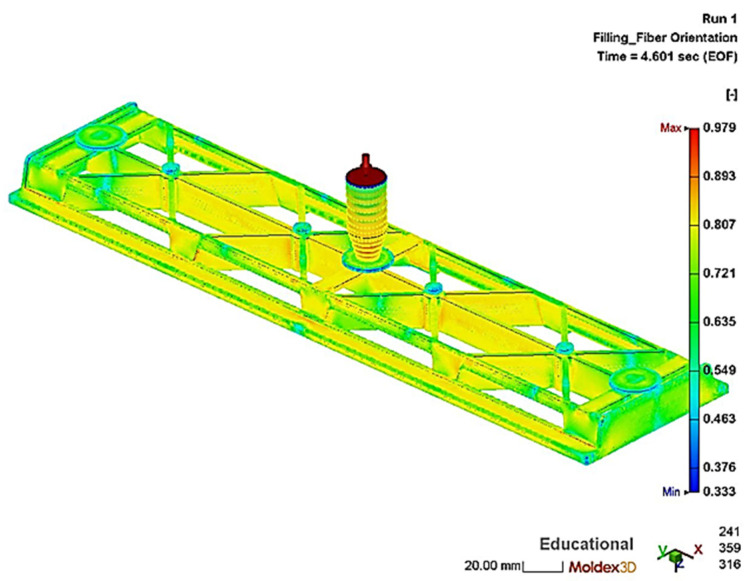
Simulated fiber orientation of the PP/GF30 short fiber-reinforced thermoplastic composite overmolding material in the hybrid test structure.

The simulations of the injection molding process with a flow speed of 50 cm^3^/s were evaluated with regard to the resulting fiber orientation and length depending on the simulation parameters. The fiber orientation corresponds to the distribution of the fiber orientation vector of the plastic melt at the end of the packing step and can be seen in Figure 9 for the hybrid injection molded structure. In here, a value of 0.33 means that the orientation of the fibers is random and a value of 1 means that the fibers are 100% oriented in the flow direction. The obtained data of the hybrid injection molding simulation was imported into ANSYS Workbench (ANSYS 2021R1, Ansys Inc., Canonsburg, PA, USA) for structural analysis. Additionally, experimental validation of the simulated local fiber length and orientation for the test plates was carried out.

#### 2.3.4. Numerical Simulation of Flexural Test

**Figure 10:**
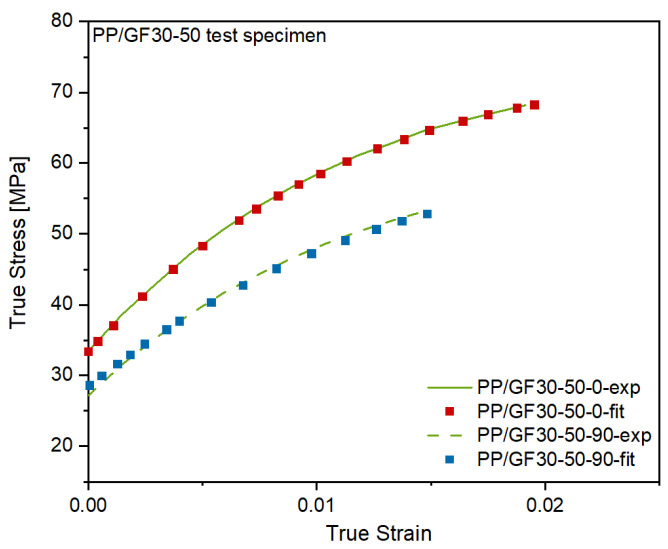
Experimental and fitted stress-strain-curves of the PP/GF30 short fiber-reinforced thermoplastic composite in flow direction and perpendicular to it.

The numerical simulation of the flexural test of the hybrid injection molded thermoplastic composite beam was carried out within ANSYS workbench 2021R1 (ANSYS 2021R1, Ansys Inc., Canonsburg, PA, USA) as a static structural simulation. The part geometry and mesh were implemented as described in the workflow of Figure 7. A proper mesh was generated by using volume elements of an edge length of 2 mm. Elements with a linear regression function were used and the mesh size and elements were checked by convergence study. In general, two types of homogenizations for material are possible, a finite element representative elementary volume model or using a mean field Mori-Tanaka analytical model [31]. In this study the analytical model was used. Therefore, the engineering data of the reinforcing fibers and of the matrix polymer was added separately in the material designer. Here, the different material properties of the polymer matrix and the reinforcing fibers were defined by directly specifying orthotropic elastic material values for both phases. A fiber volume fraction of 13.5% and an aspect ratio of 23.5 were defined for the short fiber-reinforced overmolding material. In the next step, the master curves of the tensile tests of the short fiber-reinforced thermoplastic composites were added to the material designer to define their nonlinear isotropic hardening behavior by curve fitting according to the Hill plasticity model (see Figure 10). Therefore, the experimental data of true stress and plastic strain were implemented. The final material parameters used for the material model definition are shown in Table 4.

**Figure 11:**
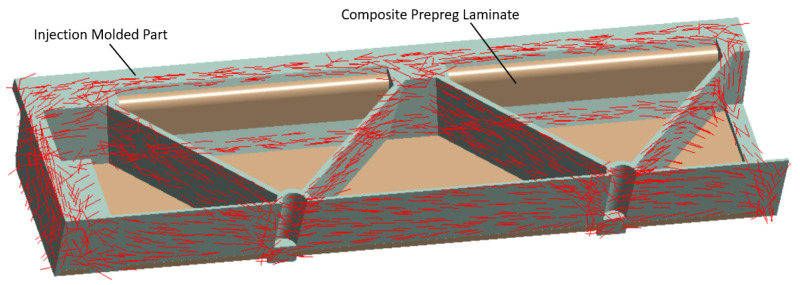
Main fiber directions of the PP/GF30 short fiber-reinforced thermoplastic composite mapped to the meshed geometry of the test bending structure using the orientation tensor output from the injection molding simulation. The injection molded part is directly bonded to the continuous fiber-reinforced laminate using contact elements.

**Figure 12:**
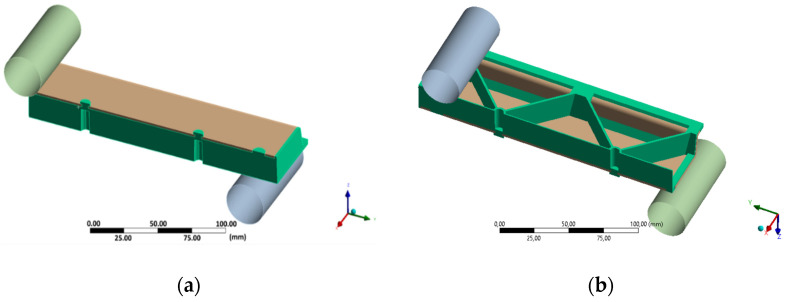
Quarter model of the three-point flexural test of the hybrid injection molded thermoplastic composite test structure. The injection molded PP/GF30 short fiber-reinforced thermoplastic composite is shown in green and the PP/GF60 continuous fiber-reinforced thermoplastic composite in brown: (**a**) top view and (**b**) bottom view.

After this step, the material designer was connected to the engineering data of the static structural analysis. In this work, the following two types of data during the mapping process onto the mesh of the short fiber reinforced plastic part were considered: the principal fiber directions from the injection molding simulations as shown in Figure 11 and the two largest eigenvalues of the fiber orientation tensor. Additionally, local variation of the nominal fiber volume fraction and the presence of residual stresses can be considered but were neglected here. The short fiber-reinforced thermoplastic composite part was then combined with the continuous fiber-reinforced laminate using contact elements to simulate a perfect bonding interface. A quarter model of the hybrid composite test structure in a three-point flexural test configuration is shown in Figure 12. In here, the injection molded short fiber-reinforced thermoplastic area is shown in green and the continuous fiber-reinforced thermoplastic in brown. The following displacements constraints were applied to the FE model: the support is imposed with no displacement as well as no rotations and the punch with a 25 mm ramped displacement in y direction.

Since the described structural model is based on an implicit calculation and it is limited for a quasi-static load case situation. The prediction of the mechanical behavior of the test structures is therefor only as good as the material model is precise and valid. Deviations between the experiment and the simulation of the test structures mainly arise because the data for the material model has been determined on standard tensile test specimens and it is assumed that these properties and hardening behavior is also found in the real test structure.

## 3. Results and Discussion

### 3.1. Anisotropy of Injection Molded Short Fiber-Reinforced Thermoplastic Composites

**Figure 13:**
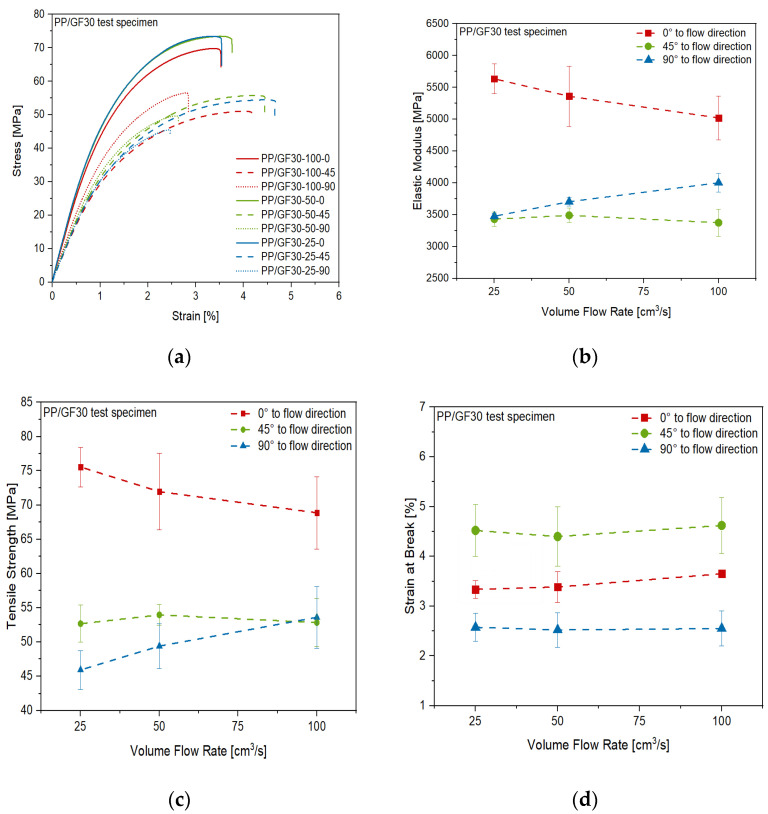
Anisotropic mechanical properties of the PP/GF30 short fiber-reinforced thermoplastic composite as a function of volume flow rate during injection molding: (**a**) stress-strain diagram from tensile test, (**b**) elastic modulus, (**c**) tensile strength and (**d**) strain at break.

The results of the tensile tests of the PP/GF30 short fiber-reinforced thermoplastic composite material are shown in Figure 13. Anisotropic and inhomogeneous behavior of the material was found, depending on the volume flow rate and direction during the injection molding process of the test plates. This can be seen in the stress-strain-curves of the test specimen cut out in the different directions (Figure 13a). The highest stiffness and strength were measured in flow direction, while these properties are decreasing with volume flow rate, which can be seen in Figure 13b,c. It is also shown that the opposite trend was found perpendicular to the flow direction, while no significant influence of the flow rate on the stiffness and strength in 45° to the flow direction was found. However, in this direction the highest strain at break was measured, without any significant influence of the flow rate, as shown in Figure 13d. These trends correlate to the flow rate dependent short fiber orientation mechanisms during injection molding as published by Bay and Tucker III [35,36]. As reported by Oumer and Mamat [37], at low flow rates the short fibers are more oriented in the flow direction and therefore the stiffness and strength of the composite are higher in this direction. Given the results shown in Figure 13, the same mechanism has to be stated for the PP/GF30 short fiber-reinforced composite material used in this study.

**Figure 14:**
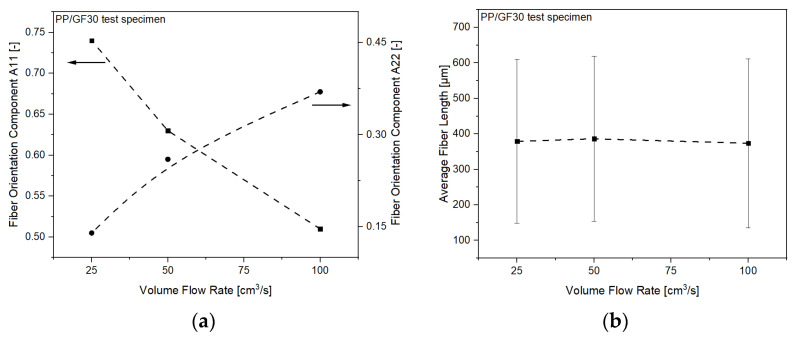
Morphological properties of the PP/GF30 short fiber-reinforced thermoplastic composite as a function of volume flow rate: (**a**) fiber orientation components A11 and A22 as experimentally measured by CT and (**b**) average fiber length as experimentally measured by fiber length analysis.

To validate this effect, CT measurements of the fiber orientation in the processed PP/GF30 short fiber-reinforced thermoplastic composite test samples have been analyzed. The measured fiber orientation components A11 and A22 of the composites are shown in Figure 14a. It can be seen that the A11 orientation component is decreasing with increasing flow rate, while the opposite trend is shown for the A22 component. This clearly indicates the flow rate dependent fiber orientation and resulting anisotropic mechanical behavior. As reported by Huang and Lai [38], this can be attributed to a flow-fiber coupling effect, diminishing the flow direction orientation tensor component A11 and simultaneously enhancing the cross-flow orientation tensor component A22. However, no influence of the flow rate on the average fiber length was found, as shown in Figure 14b. Numerical simulation of such fiber orientation mechanisms has been studied and significant theoretical orientation models have been developed, including RSC (Reduced Strain Closure), ARD (Anisotropic Rotary Diffusion), and iARD-RPR (improved ARD and Retarding Principal Rate) [39,40,41]. They have been widely applied in commercial injection molding simulation software such as Moldex3D or Moldflow, with good agreement between the predictions and the experimental data obtained.

### 3.2. Numerical Simulation and Experimental Validation of Injection Molding of Short Fiber-Reinforced Thermoplastic Composites

**Figure 15:**
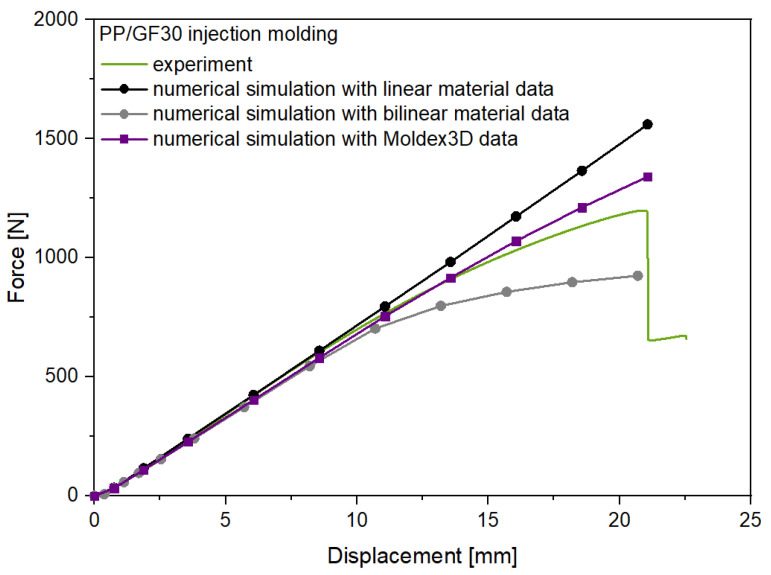
Experimental and numerical results for the flexural test of the PP/GF30 short fiber-reinforced thermoplastic composites test structure.

The experimental and numerical results for the flexural test of the PP/GF30 short fiber-reinforced thermoplastic composite test structure are shown in Figure 15. As can be seen, the experimental force-displacement curve is showing nonlinear behavior after a displacement of 10 mm. These experimental results are compared to the three different structural analysis carried out with linear, bilinear and injection molding simulation data (Moldex3D) to investigate the effect of the precise consideration of the anisotropic mechanical behavior. For the structural analysis with the linear and bilinear material approach no fiber orientation information were added from the injection molding simulation.

In result, for all three material models the initial stiffness of the injection molded short fiber-reinforced composite test structure was predicted well. However, the nonlinear displacement behavior was not predicted correctly by the approaches with the linear and bilinear material models, leading to an overprediction of the flexural strength by the linear model with a deviation of 31% and an underprediction with a deviation of 21% by the bilinear model. In contrast, the simulated force-displacement curve considering the input data from the injection molding simulation fits the experimental data significant more precisely but also not completely until failure at 21 mm displacement, leading to an overprediction of the flexural strength with a deviation of 13%. However, these findings demonstrate the importance of consideration of the process dependent anisotropic and local inhomogeneous mechanical behavior of short fiber-reinforced thermoplastic composites in the structural analysis of injection molded parts. This has also been reported for other short fiber-reinforced thermoplastic composite materials using specific anisotropic simulation methodologies. Gruber and Wartzack [42] could improve the precision of the absolute displacement prediction of short fiber-reinforced composite structures under flexural loading by usage of an integrative simulation approach similar to the numerical workflow in this study. Ogierman and Kokot [43] showed in a comparative analysis of a short fiber-reinforced composite with different fiber orientations that simplification of the material model of injection-molded parts and considering it as isotropic corresponding to the random orientation of the fibers or orthotropic corresponding to unidirectional orientation can lead to unacceptable errors in determining the natural frequencies, displacement and stresses. This was also reported by Vlach and Stekly [44], who showed that the prediction deviation of the longitudinal modulus and modal frequencies in complex shaped injection molded parts made of short fiber-reinforced composites can be significantly reduced by consideration of the anisotropic material behavior.

### 3.3. Numerical Simulation and Experimental Validation of Flexural Test of Hybrid Injection Molded Thermoplastic Composites

**Figure 16:**
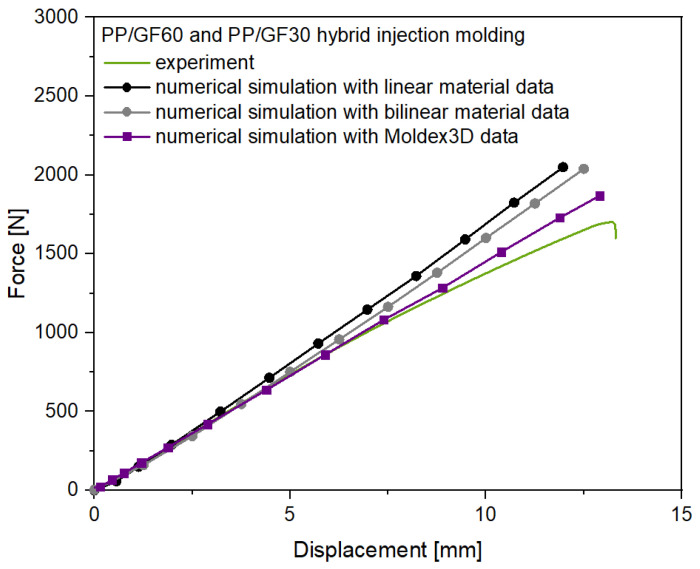
Experimental and numerical results for the flexural test of the PP/GF60 and PP/GF30 hybrid injection molded thermoplastic composite test structure.

The experimental and numerical results for the flexural test of the hybrid PP/GF60 continuous TPC and PP/GF30 short fiber-reinforced thermoplastic composite test structure are shown in Figure 16. In comparison to the short fiber-reinforced test structure without the continuous fiber-reinforced laminate the stiffness of the hybrid structure prevents a stronger nonlinear behavior and shows only little yielding effects, leading to an experimental curve nearly linear until failure. Additionally, the flexural strength of the hybrid test structure was increased in comparison to the short fiber-reinforced thermoplastic composites test structure. However, to investigate the influence of the anisotropic mechanical behavior of the short fiber-reinforced overmolding material, again three different numerical simulation were made. The approaches with a linear and additionally with a bilinear material model and with no information on fiber orientation from the injection molding simulation did not predict the deformation behavior of the hybrid test structure correctly, showing significant deviations after 5 mm displacement. Both approaches lead to an overprediction of the flexural strength by 21% and 20%, respectively. This can be explained with the dominant stiffness of the PP/GF60 laminate in the test structure. Implementation of the local fiber orientations from the injection molding simulation and using a variable short fiber-reinforced composite material model did significantly improve the accuracy of the simulation with a resulting overprediction of the flexural strength 9%. However, when the hardening behavior of the short fiber-reinforced composite material is considered the simulated force-displacement curve fits the experimental data but also not completely until failure at 13 mm displacement.

These findings show that the anisotropic and local inhomogeneous mechanical behavior of the short fiber-reinforced thermoplastic overmolding material in hybrid structures with continuous fiber-reinforced composites must be considered to predict the mechanical behavior, especially at higher flexural stresses. Comparable findings have been reported by Ding et al. [45], where the overprediction of the flexural strength was attributed to the brittleness of the composite structure, leading to penetration of the material by the load head during the test, and the delamination failure at the interfaces of the TPC material. Both effects have to be considered for the experimental results of the flexural test with the hybrid test structure in this study as well, similarly leading to an overprediction of the flexural strength by the numerical simulations. However, in comparison to the findings of Zscheyge et al. [46], who focused on the damage modeling of the TPC part of a comparable hybrid structure and considered isotropic material modeling of the overmolding part, the numerical simulation of the flexural behavior in this study are more precise. Additionally, the deviations of the simulation to the experimental results at higher displacements can be addressed to the fact that the hardening curves of the PP/GF30 short fiber-reinforced composite overmolding material were determined with the results of the tensile tests of the injection molded test plates. Here, especially the different cooling rates during processing can lead to a different crystallinity and hardening behavior in comparison to the test structure of the flexural tests [47].

## 4. Conclusions

Short fiber-reinforced thermoplastic composites processed by injection molding show anisotropic mechanical behavior, which is dependent on the local fiber orientation resulting from part design and process parameters. As shown in this study for PP/GF30 test plates, the volume flow rate during injection molding has a significant influence on the fiber orientation of such short fiber-reinforced composites with a higher orientation in flow direction and at lower volume flow rates. This results in higher stiffness and strength values in the flow direction at lower volume flow rates, causing a process dependent anisotropic mechanical behavior. Consideration of this anisotropic and local inhomogeneous mechanical behavior is important for the precise prediction of the deformation and failure behavior of injection molded parts by numerical simulations. This is shown for the deformation behavior of a PP/GF30 short fiber-reinforced test structure under flexural load with a new numerical workflow implementing the local fiber orientation as provided by injection molding simulation. In result, the deviation of the numerically predicted and experimentally measured flexural strength could be reduced from 31% to 13%. Thus, it has also been shown for a PP/GF60 and PP/GF30 hybrid injection molded thermoplastic composite test structure that consideration of the anisotropic and local inhomogeneous mechanical behavior of the overmolding material is as well crucial for prediction of the deformation behavior of such hybrid short and continuous fiber-reinforced thermoplastic composites under flexural load. The deviation of the numerically predicted and experimentally measured flexural strength could be reduced from 21% to 9% in this case. Although the continuous fiber-reinforced composites dominate the mechanical behavior of the hybrid structure at low displacement, the critical failure is prone to be initiated by crack propagation in the short fiber-reinforced overmolding material due to the higher flexural stresses at higher displacements and lower strength of this material.

## Figures and Tables

**Table 1:** Properties of the PP/GF60-UD unidirectional glass fiber-reinforced polypropylene tapes used for manufacturing of TPC laminates as provided by the supplier.

Property	Unit	Value
Glass fiber mass content	wt%	60
(Ply) thickness	mm	0.25
Density	g/cm^3^	1.5
Tensile modulus E11	GPa	28
Tensile modulus E22	GPa	3.2
Tensile strength	MPa	720
Flexural modulus	GPa	21
Flexural strength	MPa	436

**Table 2:** Properties of the PP/GF30 short fiber-reinforced thermoplastic composite used for overmolding of TPCs as provided by the supplier.

Property	Unit	Value
Glass fiber mass content	wt%	30
Glass fiber length	mm	0.5
Density	g/cm^3^	1.14
Tensile modulus	GPa	6.5
Tensile strength	MPa	90
Flexural modulus	GPa	5.5
Flexural strength	MPa	120

**Table 3:** Labeling of the analyzed test specimen of the PP/GF30 short fiber-reinforced thermoplastic composites and the corresponding volume flow rate and orientation related to flow direction due to injection molding and sample preparation from test plates, respectively.

Denomination	Volume Flow Rate [cm^3^/s]	Orientation Related to Flow Direction [°]
PP/GF30-100-0	100	0
PP/GF30-100-45	100	45
PP/GF30-100-90	100	90
PP/GF30-50-0	50	0
PP/GF30-50-45	50	45
PP/GF30-50-90	50	90
PP/GF30-25-0	25	0
PP/GF30-25-45	25	45
PP/GF30-25-90	25	90

**Table 4:** Numerical simulation input parameters of the PP/GF30 short fiber-reinforced thermoplastic composite.

Property	Unit	Value	Origin
Tensile modulus of matrix (PP)	GPa	1.4	supplier
Tensile modulus of reinforcing fiber (GF)	GPa	73	supplier
Fiber volume content	%	13.5	pyrolysis
Fiber aspect ratio	-	23.5	fiber length analysis
Fiber orientation component A11	-	0.61	CT analysis
Fiber orientation component A22	-	0.27	CT analysis
Yield strength σ_0_	MPa	20.9	ANSYS
Hardening law parameter R_0_	-	313.4	ANSYS
Hardening law parameter R_infty_	-	22.6	ANSYS
Hardening law parameter b	-	245.2	ANSYS

## Data Availability

All data included in this study are available upon request by contact with the corresponding author.
